# Plasma endotoxin core antibody concentration and linear growth are unrelated in rural Malawian children aged 2–5 years

**DOI:** 10.1186/s13104-015-1238-1

**Published:** 2015-06-24

**Authors:** Nicole Benzoni, Poonum Korpe, Chrissie Thakwalakwa, Ken Maleta, Kevin Stephenson, Micah Manary, Mark Manary

**Affiliations:** Department of Pediatrics, Washington University, St. Louis, MO 63110 USA; School of Public Health, Johns Hopkins University, Baltimore, MD USA; Department of Community Health, College of Medicine, University of Malawi, Blantyre, Malawi; College of Physicians and Surgeons, Columbia University, New York, NY USA; School of Medicine, University of California, San Diego, San Diego, CA USA; Department of Pediatrics, St. Louis Children’s Hospital, One Children’s Place, St. Louis, MO 63110 USA

**Keywords:** Endotoxin core antibody, Environmental enteropathy, Growth, Malawi

## Abstract

**Background:**

Environmental enteropathy is subclinical inflammation of the upper gastrointestinal tract associated with reduced linear growth in developing countries. Usually investigators have used biopsy or a dual sugar absorption test to assess environmental enteropathy. Such tests are time and resource intensive, restricting their utility as screening methods. Serum endotoxin core antibody (EndoCab) concentration is a potential indicator of intestinal inflammation and integrity, and thus may be useful to predict environmental enteropathy. We analyzed the association of serum EndoCab levels versus linear growth and lactulose-mannitol assay results in 2–5 year old rural Malawian children.

**Methods:**

This was an observational study of 388 rural, asymptomatic Malawian children who had anthropometric measurements taken at least every 3 months since birth. In June and July 2011, dual sugar permeability tests were performed and serum samples were drawn for EndoCab assays. Pearson correlation, Student’s t test and multivariable linear regression were used to compare ln EndoCab concentrations with height-for-age z scores (HAZ) at time of sampling and 3 months later. Identical analysis was also performed for ln EndoCab versus measurements from dual sugar permeability testing performed in conjunction with serum sampling. In a subgroup of children with anthropometric data in the months prior to serum sampling, Pearson correlation was used to estimate the relationship between ln EndoCab and recent linear growth.

**Results:**

Ln EndoCab concentrations were not correlated with HAZ at time of measurement (*B* = −0.078, *P* = 0.14) nor change in HAZ over the subsequent 3 months HAZ (*B* = −0.018, *P* = 0.27). EndoCab concentration was not associated with %lactulose excretion (*B* < 0.001, *P* = 0.98) nor the lactulose:mannitol ratio (*B* = 0.021, *P* = 0.62). Subgroup analysis also did not reveal any significant association between EndoCab and recent growth.

**Conclusion:**

EndoCab titers were not correlated with measurements of growth or intestinal permeability in rural pre-school aged Malawian children.

## Background

Stunting, defined as stature less than two standard deviations below the median height-for-age, affects an estimated one in four children worldwide [1]. Stunted children experience reductions in physical capability and school performance as well as modest compromises in immune function [2]. Randomized trials of multiple micronutrient provision, dietary supplementation and antimicrobial interventions to reduce the infectious disease burden do not substantially reduce stunting [3]. Stunting is thought to be caused by chronic malnutrition and diarrheal illness; gastrointestinal inflammation has been implicated as an important additional contributor [3–5].

Environmental enteropathy (EE) is a subclinical condition with T-cell infiltration of the duodenum and jejunum, decreased villous height and increased crypt depth [3, 6, 7]. Children with EE have increased intestinal permeability and reduced nutrient absorption. EE has been associated with the reduced linear growth and cognitive function [5, 8]. The etiology of EE is unknown [3]. Historically, diagnosis of EE was determined via small bowel biopsy revealing the aforementioned architectural changes. Biopsy is too costly and invasive to serve as a screening tool in developing countries where EE is prevalent [7].

Dual sugar absorption, or lactulose:mannitol (L:M), testing provides a less invasive measure of gut health at a single point in time [9]. Increased %lactulose (%L) excretion suggests impaired intestinal barrier function; decreased %mannitol (%M) excretion correlates with decreased functional intestinal surface area [10]. The L:M test is time and resource intensive and therefore often difficult to accomplish in settings where stunting is pervasive. The reliability and validity of L:M as a measure of intestinal health is contingent upon the clinical protocol for test administration and the analytical method used to quantitate sugar concentrations [11, 12]. Recent research has focused on developing an alternative, practical biomarker for EE [3, 11, 12].

Serum titer of endotoxin core antibody (EndoCab) showed promising results in reflecting linear growth and intestinal function among Gambian infants, prompting further analysis of EndoCab as a candidate biomarker [5, 11]. EndoCab is produced in response to the lipopolysaccharide component of gram-negative bacteria’s cell wall after systemic exposure to enteric bacteria [3]. Bacterial degradation products may translocate across the impaired gastrointestinal mucosal barrier and induce EndoCab formation. It was thus hypothesized that EndoCab levels may be a cumulative measure of EE burden in affected children [11]. Recent analyses of EndoCab versus growth and permeability revealed conflicting findings in Bangladeshi and Zimbabwean infants <2 years old and no relationship to concurrent height measurements in young Bangladeshi children [8, 13, 14]. Thus further research is required to determine the utility of EndoCab measurements, particularly in children over 2 years of age.

Among a population of 2–5 year old rural Malawian children in whom we have previously demonstrated an association between growth and %L, we assessed EndoCab levels within collected serum samples [10]. This observational study tests the hypothesis that EndoCab is inversely correlated to cumulative linear growth as well as linear growth in the time period proximate to EndoCab measurement.

## Methods

### Subjects

A total of 418 healthy children aged 2–5 years were recruited from five rural subsistence farming areas in southern Malawi in June and July of 2011 from a population enrolled in a longitudinal gut microbiome study [10, 15]. These children consumed a diet primarily of maize and legumes and had growth monitored every 2–3 months since birth. Of the original cohort, 388 children had serum samples collected for EndoCab assessment and were eligible for this analysis. Exclusion criteria included any chronic debilitating illness, including known HIV infection or obvious congenital abnormalities, evidence of acute malnutrition, and a recent history or current case of diarrhea or hematochezia. Evidence for the exclusion criteria was sought by interview, review of the child’s heath record and physical examination, including anthropometry.

Written and oral consent was obtained from every subject’s caregiver prior to participation. The study was approved by the College of Medicine Research Ethics Committee of the University of Malawi, the Washington University Human Research Protection Office, and the Baylor College of Medicine Human Investigations Review Board.

### Participation

Information regarding demographics, household hygienic practices and the child’s recent health was obtained at enrollment prior to serum collection through in-person interview with the child’s caregiver. Individual dietary diversity scores (IDDS) were assessed at baseline [16]. Anthropometric measurements throughout the study were obtained following procedures adapted from the Food and Nutrition Technical Assistance (FANTA) project [17]. Weight was measured using an electronic scale to the nearest 5 g. Length was measured to the nearest 0.2 cm using a rigid length board three separate times and the three values were averaged [17]. Age was calculated as months since the child’s birthdate, based on each child’s Malawian Health Passport, an official document issued by the Malawian government containing the child’s date of birth and vaccination record [18]. Approximately 3 months after the initial visit, a second set of anthropometric measurements and a survey of recent child health were collected.

A dual sugar absorption test was conducted at the initial visit to assess intestinal function and integrity. Caregivers were directed not to feed the child for 12 hours prior to participation during a preparatory meeting, and asked to come to the study site before 6 am. Children were encouraged to drink water to facilitate urination and mothers were instructed not to breastfeed their children during this time. Children were given the non-metabolized sugars lactulose (5 g) and mannitol (1 g) dissolved in 100 mL of water. A complete 4 h urine collection was made and 10 mg merthiolate was added to the urine collection cup to limit bacterial degradation of the sugars. The total urine volume was measured and a 4 mL aliquot was transferred to a cryovial, flash frozen in liquid nitrogen, and transported at −80°C to Baylor College of Medicine.

A serum sample was drawn during the dual sugar absorption testing, transferred to a clot activator tube, kept cool on ice while the children completed their urine collection, and then spun at 3,000 rpm for 15 min. Serum was transferred to a clean cryovial and frozen in a −80°C freezer. These sera were transferred to the University of Virginia, Charlottesville, VA as a batch for EndoCab analyses.

### Laboratory analyses

Concentrations of lactulose and mannitol in the urine specimens were analyzed by high pressure liquid chromatography as has been previously described [19]. The assays are sensitive to 1 μg/mL lactulose and mannitol and the coefficient of variation (CV) for this sample set was ≤5% [19].

Serum was tested using a commercially available EndoCab IgG ELISA assay (Hycult Biotech Inc., The Netherlands) chosen from the same lot and purchased at the same time. Prior to testing, serum samples were brought to room temperature and the assay was performed per package insert. Samples were run in duplicate and mean absorbance values measured using a Biotek Synergy H4 Plate Reader. A standard curve with good curve fit was generated using Gen5 software (BioTek Instruments, Inc. USA).

### Data analyses

Data were double entered into a Microsoft Access database and cleaned by correcting any discrepancies identified by double entry, using original collection forms as reference. Anthropometric indices based on the World Health Organization’s 2006 Child Growth Standards were calculated using Anthro v 3.1 (WHO, Geneva). Linear growth was defined as the difference between length at serum sampling and at follow-up approximately 3 months later. All analyses were conducted using SAS System for Windows, Version 9.4 (SAS Institute Inc. Cary, NC, USA). Differences were considered statistically significant at *P* < 0.05, unless otherwise noted.

Urinary excretion of lactulose and mannitol (as percentage of dose administered) and L:M ratio were calculated. Similar to previous analyses, an L:M ratio >0.10 was defined as abnormal, based on reference values from healthy children in developed countries [20]. Normality was assessed for continuous variables using Shapiro–Wilk test, variables with *P* > 0.05 were considered normally distributed [21]. Variables with non-normal distribution, including EndoCab, %L and L:M values, were approximated to a normal distribution by log transformation.

Anthropometric and permeability measures were divided approximately into quartiles to compare mean ln EndoCab levels amongst children with similar health metrics. Children were divided into quartiles defined by (1) height-for-age z score (HAZ), (2) change in HAZ, (3) L:M ratio and (4) %L excretion. For each set, one-way analysis of variance for interquartile mean ln EndoCab was conducted using the Tukey–Kramer HSD test [22].

Bivariate parametric correlation tests were conducted to identify statistically significant predictors of linear growth and intestinal permeability. Pearson correlation testing was used to compare the height and permeability measures with continuous variables and Student’s t test to compare with binary variables. Separate analyses were performed for four dependent continuous variables: (1) HAZ at the time of serum sampling, (2) change in HAZ 3 months later, (3) ln L:M ratio and (4) ln %L excretion.

To determine ln EndoCab’s ability to predict linear growth after taking into account other factors, backward multivariable linear regression modeling was conducted. Independent variables with a Pearson product-moment correlation coefficient or t-statistic with an associated *P* value <0.20 were eligible for inclusion in each respective model. Other independent predictor variables considered for inclusion were dietary diversity, sanitation and socioeconomic characteristics collected at baseline and previously considered during regression modeling in the overall Malawian cohort [10]. These included the child’s age in months at serum sampling, gender, family ownership of a bicycle, whether the child’s house had a metal roof and previous treatment with therapeutic food. Additional anthropometric measurements measured at serum sampling, mid-upper arm circumference Z-score (MUACZ) and weight-for-age Z-score (WAZ). were only considered in the model for change in HAZ over 3 months. As the primary variable of interest, ln EndoCab was included regardless of statistical significance. Multicollinearity testing was conducted by measuring variance inflation factor (VIF) amongst assessed variables. A VIF >5 was considered suggestive of multicollinearity. Variables were eliminated from the model if *P* > 0.05.

A subgroup analysis was undertaken to investigate a possible relationship between ln EndoCab and linear growth in the months prior to antibody measurement, which would suggest that serum EndoCab reflects previous growth trends. Children were eligible for inclusion in the subgroup if anthropometric data was available for the 6 months preceding serum sampling. Pearson correlation analysis was performed between HAZ or change in HAZ (from 3 to 6 months prior to serum sampling) and ln EndoCab concentration.

## Results

Of the 388 children included in this analysis, 301 (78%) were stunted and 340 (88%) had an L:M ratio suggestive of EE (Table 1). The average EndoCab concentration was 74.2 GMU/mL, nearly twice the mean expected titer for children with normal intestinal absorption and barrier function, based on titers from healthy UK children [5]. No statistically significant difference was observed in average ln EndoCab concentrations amongst children stratified into quartiles by (1) HAZ at serum sampling, (2) change in HAZ over 3 months, (3) L:M ratio or (4) %L (Tukey–Kramer test) [22].

**Table 1:** Characteristics of rural Malawian children enrolled in EndoCab analysis, n = 388

Characteristic	Mean (±SD) or N (%)
Age, months	40.5 (±8.6)
Male	182 (47%)
Endotoxin antibody, GMU/mL	74.2 (±100.7)
Height-for-age, z score	−2.80 (±1.1)
Change in HAZ 3 months after EndoCab analyses	0.3 (±0.3)
Weight-for-age, z score	−1.5 (±0.9)
Weight-for-height, z score	0.3 (±0.9)
%Lactulose	0.3 (±0.2)
%Mannitol	6.6 (±5.4)
Lactulose:mannitol	0.2 (±0.2)
L:M ≥0.10	340 (88%)
L:M <0.10	48 (12%)
House has a metal roof	102 (26%)
Child cleanses hands with soap after stooling	192 (50%)
Child previously treated for malnutrition	12 (4%)

Pearson product-moment correlation coefficients were calculated to estimate the relationship between ln EndoCab and linear growth as well as dual sugar absorption tests (Table 2). There was no statistically significant association between ln EndoCab and HAZ (*r* = −0.048, *P* = 0.35) or change in HAZ (*r* = −0.078, *P* = 0.12). Comparison of permeability measures to ln EndoCab revealed no significant relationship with either ln %L (*r* < 0.001, *P* = 0.99) or ln L:M (*r* = 0.025, *P* = 0.64).

**Table 2:** Pearson correlation and regression models predicting HAZ, change in HAZ over 3 months and intestinal permeability markers using serum ln EndoCab, n = 388

	r	*B* (SE)	P value^†^
Height-for-age, Z-score	−0.047	−0.078 (0.05)	0.14
Change in height-for-age, Z score^a^	−0.078	−0.019 (0.02)	0.27
%Lactulose	0.025	<0.001 (0.06)	0.98
Lactulose:mannitol	<0.001	0.021 (0.04)	0.62

Variables included in the final models: HAZ, adj R^2^ = 0.09 (age in mos at serum sampling, bike ownership, metal roof on house, mother as primary caretaker, long distance to water, soap use); change in HAZ, adj R^2^ = 0.02 (age in mos at sampling, initial WAZ, IDDS, bike ownership, animals sleep in house); ln %L, adj R^2^ = 0.01 (male gender, pit latrine use); ln L:M, adj R^2^ = 0.11 (age in mos at sampling).

r, Pearson product-moment correlation coefficient for ln EndoCab; *B*, standardized regression coefficient (change in respective measurement per 1-unit change ln EndoCab, adjusting for other co-variables); SE, standard error.

^†^P value associated with *B* for ln EndoCab.

^a^Change in HAZ from time of sampling to 3 months later.

After multivariable regression modeling, ln EndoCab concentration was not found to be a significant predictor of HAZ (*B* = −0.078, *P* = 0.14) or change in HAZ (*B* = −0.018, *P* = 0.27) as shown in Table 2. Thus a 1-unit increase ln EndoCab is associated with 0.078 SDs decrease in HAZ and 0.018 SDs decrease in change in HAZ over 3 months, after adjusting for other variables within the model. These changes did not reach statistical significance. Neither was ln EndoCab predictive of measures of gut integrity and permeability, either via ln %L (*B* < 0.001, *P* = 0.98) or ln L:M (*B* = 0.021, *P* = 0.62). Multicollinearity testing suggested very low levels of collinearity existed between variables (VIF <5). Covariates found to be statistically significant in each final model are presented as a footnote to Table 2.

After excluding children with less than 6 months of anthropometric data prior to serum sampling, a subgroup of 103 children remained to analyze a correlation between ln EndoCab and recent growth. This subgroup of children were on average younger (31.5 ± 3.5 mos), had higher EndoCab levels (79.6 ± 135.6 GMU/mL) and slightly lower HAZ (−3.0 ± 1.0) compared to the larger study population. No relationship was found between ln EndoCab and linear growth from either 3 or 6 months prior to its measurement (Pearson Correlation, Table 3).

**Table 3:** Subgroup characteristics and Pearson correlation coefficients for ln EndoCab concentration versus linear growth prior to serum sample collection, n = 103

	HAZ (mean ± SD)	r	P value	Δ HAZ^a^ (mean ± SD)	r	P value
At sampling	−3.01 ± 0.95	−0.068	0.49	–	–	–
3 months prior	−3.01 ± 1.97	−0.017	0.54	0.004 ± 0.36	−0.017	0.86
6 months prior	−3.04 ± 1.07	0.010	0.92	0.04 ± 0.44	−0.17	0.09

r, Pearson product-moment correlation coefficient for HAZ or Δ HAZ versus ln EndoCab.

^a^Change in HAZ from time of serum sampling compared to 3 or 6 months prior.

## Discussion

In a group of rural, immunocompetent Malawian children aged 2–5 years, EndoCab was not found to be associated with preceding or future linear growth, nor was it associated with intestinal permeability measurements. An association between the urinary %L at and linear growth was previously demonstrated in this same study population [10]. The magnitude of the titers of EndoCab from Malawian children and children in the United Kingdom suggest, but do not prove, that there is more exposure in Malawi to enteric bacteria than in the United Kingdom. The testing conditions and laboratory methods were not standardized in these two observations, so a definitive comparison cannot be made.

An intact gastrointestinal tract provides an essential barrier between a child and his or her intestinal microbes. Damage to its integrity decreases a child’s ability to prevent systemic exposure to inflammatory bacterial byproducts [3]. It has been hypothesized that serum EndoCab concentration may reflect cumulative gastrointestinal insult, and therefore the burden of EE, over a child’s lifetime [5, 11]. Our data analysis does not support the notion that, in 2–5 year old rural African children, EndoCab concentration reflects the lifetime or recent severity of EE. Our study is limited, however, in that EndoCab was measured at a single point in time. Since EndoCab is thought to represent chronic intestinal damage, a single IgG measurement may limit comparability to L:M or %L, which are point estimates of intestinal permeability. The utility of EndoCab may have been greater if IgM, an acute phase reactant, was measured, or if multiple measurements had been made.

Increased EndoCab levels suggest a systemic inflammatory response, which has been shown to limit linear growth in children [7]. Previous analyses of Gambian and Bangladeshi infants <2 years old identified an association between EndoCab and HAZ, but a case–control study in stunted Zimbabwean infants <18 months old found no such correlation [5, 13, 14]. Our study enrolled children between 2 and 5 years of age, therefore limiting comparability to analyses of younger children. In a study performed in older Bangladeshi children (1–4 years of age) analysis of concurrent HAZ and EndoCab measurements revealed no significant relationship [8]. The positive correlation found in some studies of younger children may be due to a temporal relationship between antibody production and exposure to enteric antigens. Younger children have had fewer exposures to endotoxin and thus each exposure, in the setting of impaired intestinal barrier in EE, results in increased EndoCab production. Older children may not exhibit changes in EndoCab concentration unless there is a substantial additional insult to the barrier function of the gut.

Discerning reasons for differences across is complicated. Recent changes in the production of the EndoCab ELISA assay limits comparability between initial and current analyses [11]. The coefficient of variation (CV) was also not assessed in this study, thus it is difficult quantify sample dispersion within the EndoCab assay. Nor has CV consistently been reported in previous articles. Intra-assay and inter-assay CVs need to be analyzed to ensure that measured serum EndoCab concentration can be compared within and between studies. Finally, the assay itself has limitations as a potential biomarker and screening tool. Serum samples for EndoCab can be collected relatively quickly, an advantage over the time intensive L:M assay, but antibody measurement requires the collection, storage and separation of blood, which is cumbersome and invasive in the setting where EE is prevalent. The generalizability of our findings are limited to rural African children, care should be taken to extrapolate these findings urban children and to regions outside Africa.

## Conclusion

The results of this study demonstrate that EndoCab is not a sensitive biomarker of either linear growth or intestinal permeability in rural African children between 2 and 5 years of age, suggesting it is not a good biomarker of EE beyond infancy in this population. Research focus may shift towards consideration of other potential EE biomarkers in older children that reflect immune response to enteric antigens, such as antiflagellin antibody, soluble CD14 or intestinal fatty acid binding protein [23, 24].

## Abbreviations

EE: environmental enteropathy
EndoCab: endotoxin core antibody
GMU: IgG standard median units
HAZ: height-for-age z score
%L: percent lactulose
L:M: lactulose mannitol ratio
